# Maternal Genetic Composition of a Medieval Population from a Hungarian-Slavic Contact Zone in Central Europe

**DOI:** 10.1371/journal.pone.0151206

**Published:** 2016-03-10

**Authors:** Veronika Csákyová, Anna Szécsényi-Nagy, Aranka Csősz, Melinda Nagy, Gabriel Fusek, Péter Langó, Miroslav Bauer, Balázs Gusztáv Mende, Pavol Makovický, Mária Bauerová

**Affiliations:** 1 Department of Botany and Genetics, Faculty of Natural Sciences, Constantine the Philosopher University in Nitra, Nitra, Slovakia; 2 Laboratory of Archaeogenetics, Institute of Archaeology, Research Centre for the Humanities, Hungarian Academy of Sciences, Budapest, Hungary; 3 Department of Biology, Faculty of Education, J. Selye University in Komárno, Komárno, Slovakia; 4 Institute of Archaeology, Slovak Academy of Sciences, Nitra, Slovakia; 5 Institute of Archaeology, Research Centre for the Humanities, Hungarian Academy of Sciences, Budapest, Hungary; 6 Research Institute for Animal Production, NAFC, Nitra, Slovakia; University of York, UNITED KINGDOM

## Abstract

The genetic composition of the medieval populations of Central Europe has been poorly investigated to date. In particular, the region of modern-day Slovakia is a blank spot in archaeogenetic research. This paper reports the study of mitochondrial DNA (mtDNA) in ancient samples from the 9^th^–12^th^ centuries originating from the cemeteries discovered in Nitra-Šindolka and Čakajovce, located in western Slovakia (Central Europe). This geographical region is interesting to study because its medieval multi-ethnic population lived in the so-called contact zone of the territory of the Great Moravian and later Hungarian state formations. We described 16 different mtDNA haplotypes in 19 individuals, which belong to the most widespread European mtDNA haplogroups: H, J, T, U and R0. Using comparative statistical and population genetic analyses, we showed the differentiation of the European gene pool in the medieval period. We also demonstrated the heterogeneous genetic characteristics of the investigated population and its affinity to the populations of modern Europe.

## Introduction

The territory of Central Europe–including the Carpathian Basin–was a place of great migration events of populations in the past. Hungarians arrived into Central Europe from across the Carpathian Mountains and settled in the Danubian Basin in 895–896 AD [1,2]. This region had been settled before the Hungarians’ arrival by Dacians, Celts, Romans, Sarmatians, Huns, German tribes (Goths, Gepids, Lombards), Avars and others, but the majority of the indigenous population was Slavic. The arrival of the Slavs and their settlement on the outskirts of the Carpathian Basin had already begun in the late migration period (6^th^ century) during which they came into contact with Lombards and Gepids [3]. At present, vast territories of East-Central and South-Eastern Europe are inhabited by Slavic populations [4]. Three groups of present-day Slavs are identified based on their linguistic affinities: Western Slavs (Poles, Czechs and Slovaks), Eastern Slavs (Ukrainians, Belarusians and Russians) and Southern Slavs (Croatians, Bulgarians, Slovenians, Bosnians, Macedonians, Montenegrins and Serbians) [5]. The modern Hungarian population, with a different cultural, linguistic and geographic origin, possesses a central location between these groups of Slavs.

**Fig 1:**
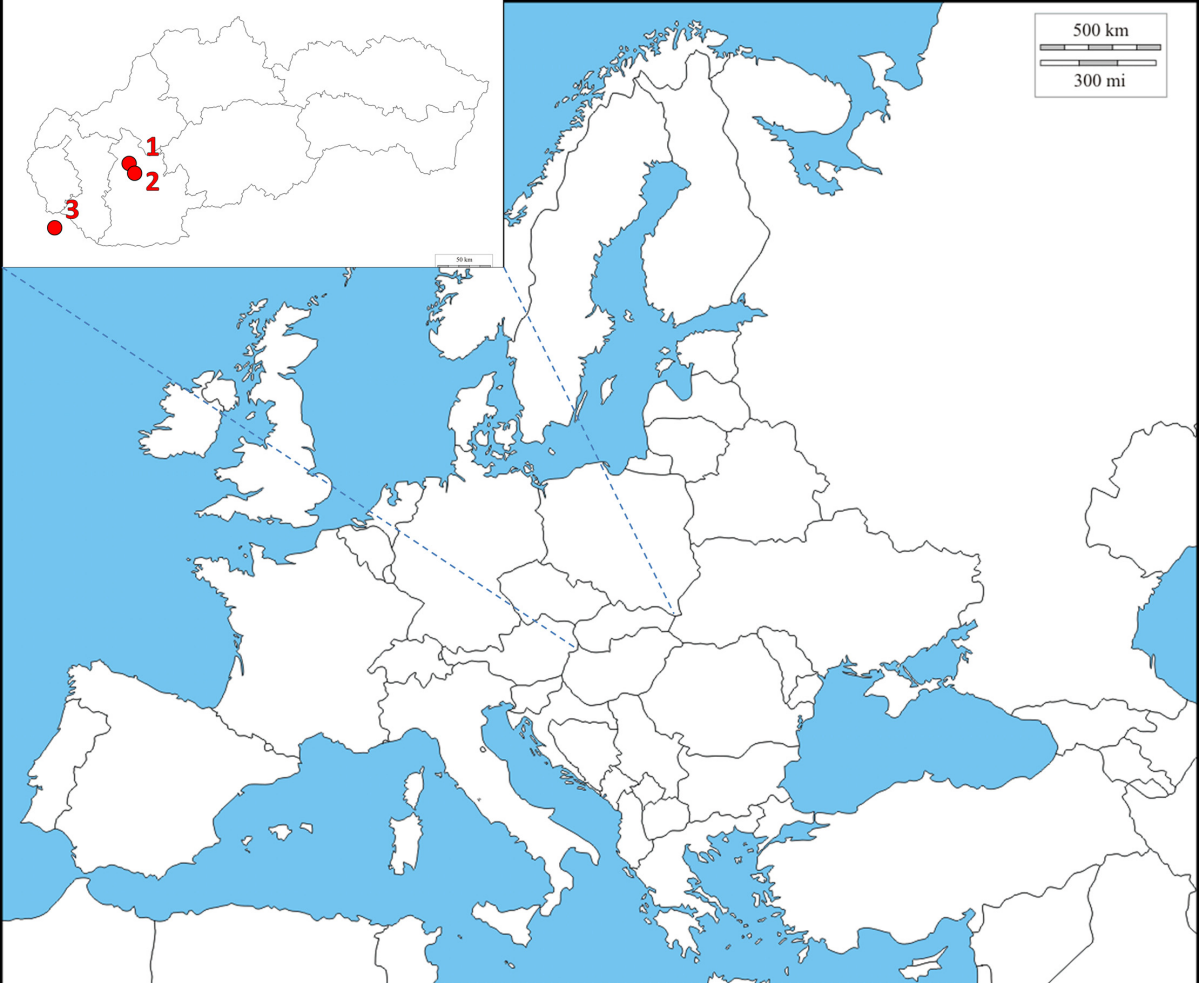
Location of medieval cemeteries from the contact zone of Central Europe. (1) Čakajovce (Slovakia). (2) Nitra-Šindolka (Slovakia). (3) Lébény (Hungary). (Illustration).

Archaeologists commonly associate certain grave goods, settlement patterns and burial customs of the cemeteries investigated at Nitra-Šindolka and Čakajovce as typically medieval plebeian mixed populations (so-called “Bijelo Brdo culture” [6,7]) of medieval Slavs and Hungarians (Magyars). The Nitra-Šindolka cemetery, dated to the 10^th^–11^th^ centuries and located in western Slovakia (Fig 1), was excavated in 1985–1986 during the construction of the highway around the town of Nitra and consists of two parts [8]. The medieval Čakajovce cemetery originating in the 9^th^–12^th^ centuries was excavated in 1974 and 1976–1986 [9].

The observed archaeological material culture is open to widely different interpretations, and in particular it is unclear whether and to what extent the attributes (e.g. grave goods and burial traditions) are indicators of ethnic and social identity, and which of these materials are linked to population movements [10]. Ancient DNA provides direct genetic evidence for past demographic events. Mitochondrial DNA (mtDNA) is a convenient tool for studying the migration of populations in the past (e.g. [11–16]) since due to its exclusive maternal inheritance it does not recombine [17], so we can monitor the mutations which have accumulated over the centuries. Therefore, human mtDNA contains a molecular recording of the genealogical history and of the migrations of women who transmitted mitochondria through the generations [15]. This information obtained from parallel biological and genetic analysis, together with archaeological and historical information and interpretations, helps us to better understand the history of human populations, their origins, migration patterns and family relationships.

The European medieval period is poorly investigated genetically; only a few hundred data have been published for the 6^th^–16^th^ centuries from Hungary, Poland, Italy, Spain and Scandinavia [10,18–25]. Furthermore, this dataset encompasses a thousand years, and is characterized by migrations and admixture of populations, by the depopulating effects of the Black Death, as well as by formations of new medieval states and ethnicities. Most of the medieval populations were heterogeneous and shared common West Eurasian mtDNA haplogroups, independent of their geographical locations and origin [10,18–25]. Consequently, more genetic data is needed to understand migration and to reconstruct the genetic composition of populations living in Europe a thousand years ago.

The aim of our study was to determine the genetic composition of maternal lineages in ancient populations from the medieval period of present-day Slovakia, and to characterize relationships to other medieval and modern human populations from Europe and Asia, using population genetics analysis. Genetic exploration of medieval populations in Central Europe is of paramount importance, because people from mixed populations of different ethnic origins lived contemporaneously in this area, making it difficult to distinguish them through archaeological records.

## Materials and Methods

### Sample information, ancient DNA extraction and amplification

The human skeletal remains (bones and teeth) used in this study were provided by the Institute of Archaeology of the Slovak Academy of Sciences in Nitra, and some of them are stored at the Slovak National Museum in Bratislava. Twenty-eight bone specimens were collected: 20 from Nitra-Šindolka and eight from Čakajovce (Fig 1). Burial sites and bones were archaeologically and osteologically processed before analysis (see S1 Table).

Sampling was carried out using gloves, facemasks and body suits to minimize the risk of contamination from modern humans. Two bone fragments, usually one tooth and one compact bone fragment of a femur, or two compact bone tissues from different parts of long bones, were collected from each individual. All stages of the work were performed under sterile conditions in a dedicated ancient DNA (aDNA) laboratory (Laboratory of Archaeogenetics in the Institute of Archaeology, Research Centre for the Humanities, Hungarian Academy of Sciences) following well-established aDNA workflow protocols [26,27]. Laboratory rooms for pre-PCR and post-PCR works are strictly separated. The laboratory work was carried out wearing clean overalls, facemasks and face-shields, gloves and over-shoes. All appliances, containers and work areas were cleaned and irradiated with UV-C light. All steps (bone cutting, surface removal, powdering, extraction, PCR set-up and amplification) were carried out in separate places. In order to detect possible contamination by exogenous DNA, one extraction and amplification blank for every five samples was used as a negative control. Haplotypes of all persons involved in processing the samples in the laboratory were determined and compared with the results obtained from the ancient bone samples (S10 Table).

The specimens were prepared following the protocols described by Kalmár et al. [28] and Shapiro and Hofreiter [29]. The bone and tooth samples were irradiated with UV-C light (1.0 J/cm^2^, 25 min). The surfaces of tooth samples were cleaned by sandblasting (Bego, EasyBlast), while the surfaces of bone samples were removed with a fresh drilling bit at slow speed, followed by UV exposure for 20 min on each side. Bone and tooth pieces were mechanically ground into fine powder in a sterile mixer mill (Retsch MM301).

Standard DNA extraction methods were used as described by Tömöry et al. [18] and Kalmár et al. [28] with some modifications. Before isolation, the samples (250 mg bone powder) were washed with 8 ml EDTA (0.5 M, pH = 7.5) overnight at room temperature with continuous vertical rotation. After centrifugation, the supernatant was discarded and the remaining samples were suspended in 1.6 ml digestion mix (0.1 M EDTA, 20% N-lauryl sarcosine and 20 mg/ml proteinase K) and incubated overnight at 37°C with continuous vertical rotation. Next day the samples were centrifuged at 13,000 rpm for 10 min, 350 μl supernatant was transferred to a fresh tube, 350 μl NH_4_-acetate (4 M) and 700 μl absolute ethanol were added, and samples were incubated overnight at −20°C. The DNeasy Tissue Kit (Qiagen) was used for further purification of the aDNA extract following the manufacturer’s instructions: the mixture was transferred into a DNeasy Mini spin column and centrifuged at 6500 rpm for 1 min. The column was washed twice, DNA was eluted in a final volume of 70 μl and subsequently stored in the pre-PCR lab at −20°C.

Several fragments of mtDNA hypervariable region I (HVR I) and the coding region were amplified in a total volume of 40 μl reaction mix, containing 6 μl of DNA extract, 20.4 μl H_2_O, 1 × AmpliTaq Gold buffer, 0.8 mM dNTP mix, 0.9 mM MgCl_2_, 0.16 mg/ml BSA, 0.625 μM primers and 1.5 U AmpliTaq Gold DNA polymerase. The HVR I region of mtDNA was amplified in two overlapping fragments with two sets of primers, and an additional six primer pairs were used to amplify haplogroup diagnostic nucleotide positions in the coding region (see S3 Table). The PCR reactions were performed in 38 amplification cycles consisting of three steps (denaturation at 94°C for 30 s, annealing at 55°C for 1 min and extension at 72°C for 30 s) with an initial denaturing step at 95°C for 10 min and final elongation at 72°C for 5 min. PCR products were checked on 8% native polyacrylamide gel. The purification of PCR products was performed using a QIAquick® PCR Purification Kit (Qiagen) following the manufacturer’s protocol. Sequencing reactions were performed using the ABI PRISM BigDye® Terminator v3.1 Cycle Sequencing Ready Reaction Kit (Applied Biosystems) and sequencing products were purified by ethanol precipitation. The sequences were determined on an ABI PRISM 3100 (PE Applied Biosystems) in cooperation with BIOMI (Budapest, Hungary). The sequences were evaluated with Chromas Lite 2.4.1 and GeneDoc [30].

The sequence polymorphisms in mtDNA (the minimum range of HVR I of samples from Nitra-Šindolka is nucleotide positions (np) 16059–16421 and the maximum range is np 16041–16421; from Čakajovce, all of them are np 16040–16400) were compared with the revised Cambridge Reference Sequence (rCRS) [31] as well as the Reconstructed Sapiens Reference Sequence (RSRS, www.mtdnacommunity.org) [32]. Haplogroup determination was carried out according to the mtDNA phylogeny of PhyloTree build 16, accessed on 19 February 2014 (www.phylotree.org) considering polymorphic positions of HVR I and six haplogroup-diagnostic position of the coding region [33]. Obtained haplotypes and defined haplogroups were proofed and compared in EMPOP database (http://empop.online/) [34]. Sequences were submitted to NCBI GenBank under the accession numbers KU739137 –KU739155.

### Reference datasets

One sample from the medieval cemetery at Lébény (Hungary), dating from the same era (Fig 1) and described by Tömöry et al. [18], was included in our analysis (S2 Table).

aDNA sequences were compared with a dataset of 24,096 present-day mtDNA sequences of the HVR I region including European, Near Eastern and Asian populations, as well as 400 medieval sequences. The sequences were collected from published data and are listed in S4 Table. Only those modern mtDNA sequences which were reported for the same sequence range as our ancient HVR I data were included in the comparison. We compared our medieval population with 11 ancient datasets, of which 10 originated from Europe and one from Asia.

### Population genetics analysis

Population comparison was calculated using Arlequin 3.5.1 [35]. Pairwise population differentiation values (F_ST_) were calculated based on HVR I sequences (np 16050–16383) assuming a Tamura & Nei substitution model [36] with a gamma value of 0.325. Significant variations in F_ST_ values were tested by 10,000 permutations between 32 modern and 12 medieval populations and particularly between the medieval populations alone (S5 and S6 Tables).

**Fig 2:**
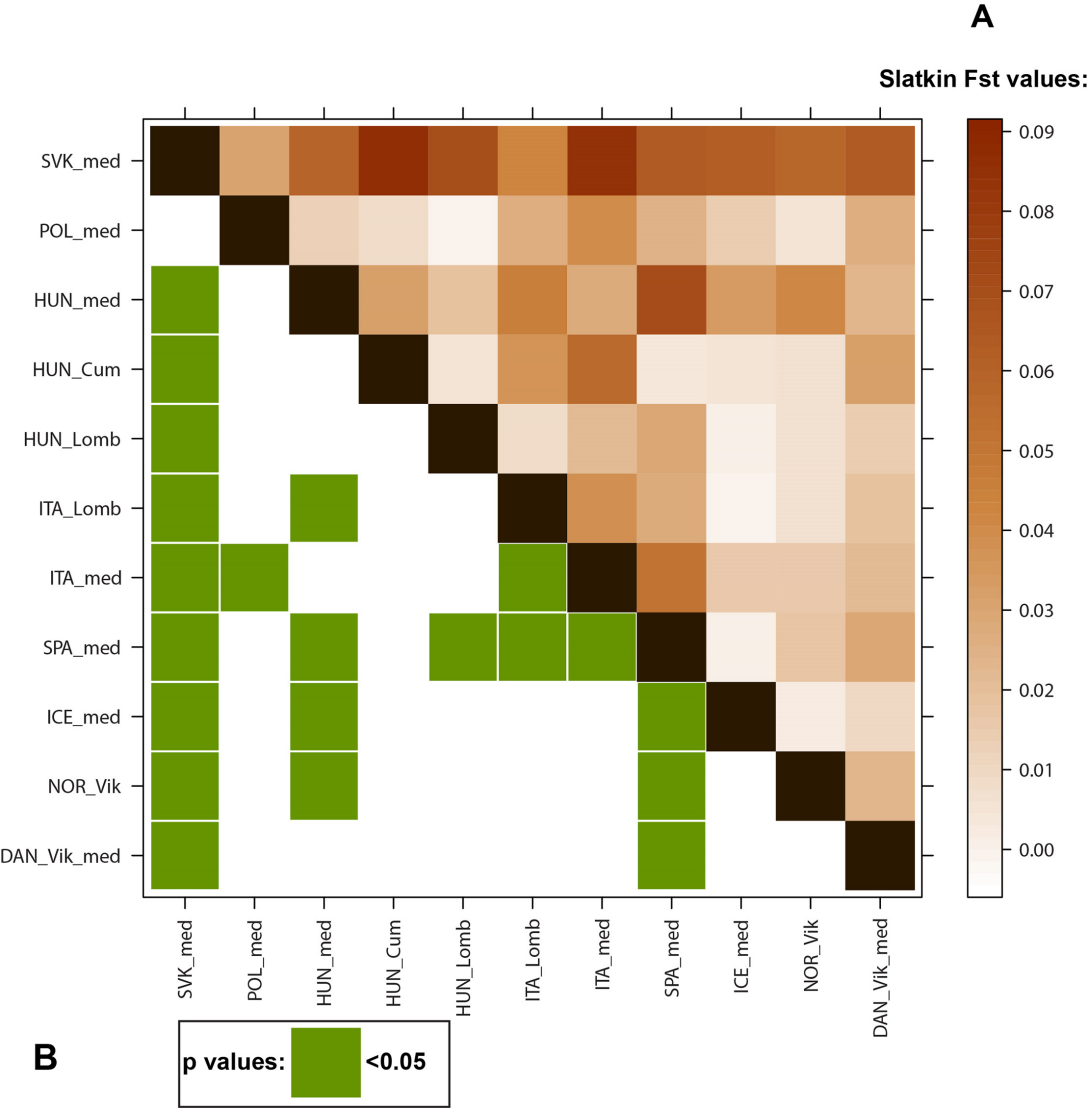
Levelplot of the linearized Slatkin population differentiation (F_ST_) values and significant p values. (A) Levelplot of the linearized Slatkin F_ST_ values of the European medieval populations. (B) significant p values (< 0.05) are indicated in green. The exact F_ST_ and p values and population information can be found in S6 Table.

Multidimensional scaling (MDS) of medieval and modern populations was applied on the matrix of linearized Slatkin F_ST_ values [37] (S5 Table) and visualized in a three-dimensional space (S1 Fig) using the metaMDS function based on Euclidean distances implemented in the Vega library of R 3.0.3 [38]. The linearized Slatkin F_ST_ values of the medieval populations were visualized on a levelplot (Fig 2).

**Fig 3:**
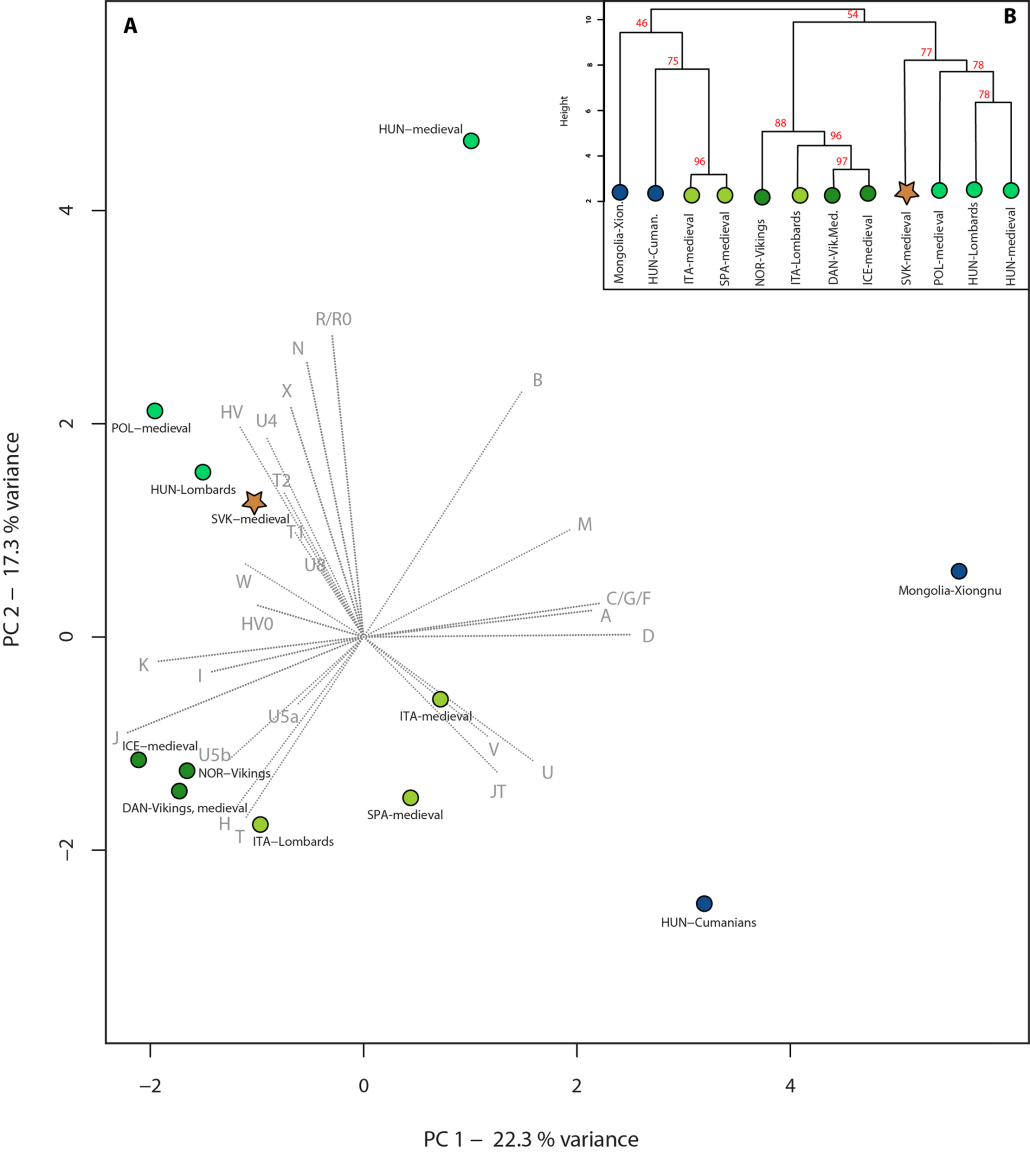
PCA and hierarchical clustering of medieval populations. PCA (A) and hierarchical clustering (B) based on mtDNA haplogroup frequencies of 12 medieval populations show a clustering of medieval populations from Slovakia (SVK-medieval), Lombards from Hungary (HUN-Lombards) and a medieval population from Poland (POL-medieval). The medieval populations and the Vikings of North Europe are clustered together, as are the medieval populations from South Europe. The index of abbreviations is in S7 Table.

**Fig 4:**
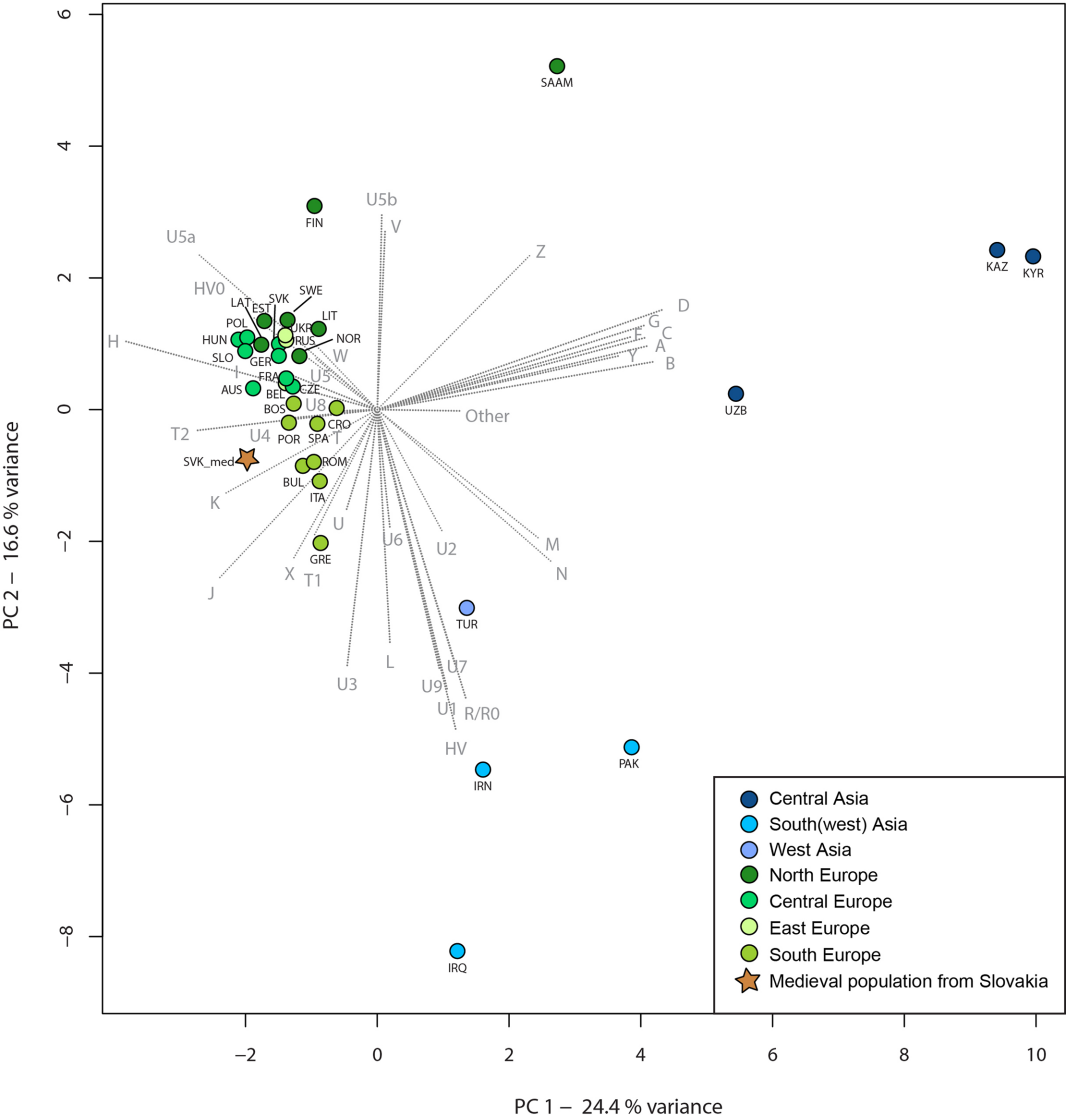
PCA of the investigated medieval and modern-day populations. The PCA is based on mtDNA haplogroup frequencies of the medieval population from Slovakia and 33 modern-day populations from Eurasia, and represents PC1 and PC2. For the frequencies and the list of abbreviations see S8 Table.

Principal component analysis (PCA) was performed, based on mtDNA haplogroup frequencies of 12 medieval and 33 modern-day populations (S7 and S8 Tables). In the PCA of medieval populations, we considered 25 mtDNA haplogroups (A, B, C/G/F, D, H, HV, HV0, I, J, JT, K, M, N, R/R0, T, T1, T2, U, U4, U5a, U5b, U8, V, W, X), whereas in PCA with modern-day populations 37 mtDNA haplogroups (A, B, C, D, F, G, H, HV, HV0, I, J, K, L, M, N, R/R0, T, T1, T2, U, U1, U2, U3, U4, U5, U5a, U5b, U6, U7, U8, U9, V, W, X, Y, Z, Other–all remaining haplogroups) were considered (Figs 3A, 4 and S2). All PCA was performed using the prcomp function for categorical PCA, implemented in R 3.0.2 [38] and plotted in a two-dimensional (prehistoric PCA) space, displaying the first two or the second and third principal components (PCs), respectively.

Besides the medieval PCA, hierarchical clustering was done using the Ward type algorithm [39] and Euclidean measurement method. All PCs (genetic variations) were used for the clustering. The result was visualized as a dendrogram with the function in R 2.13.1 [38] (Fig 3B). Cluster significance was evaluated by 10,000 bootstrap replicates using the pvclust function in R 2.13.1. The significance of each cluster was given as an AU (approximately unbiased) p value, as a percentage (Fig 3).

Shared haplotype analysis (SHA) was performed in order to detect and compare the mtDNA haplotypes shared between 12 medieval populations. Eleven populations from Europe and one from Asia were studied, and the absolute and relative shared haplotypes were counted (S9 Table).

## Results

We obtained mtDNA sequences from 19 of 28 analyzed samples originating from the medieval Nitra-Šindolka and Čakajovce cemeteries (western Slovakia). We could not extract DNA from four samples, and we excluded a further three samples from Nitra-Šindolka and one sample from Čakajovce from the statistical analysis because of bad DNA preservation or ambiguous haplotype results.

The HVR I sequences spanned the range of minimum np 16059–16400 and maximum np 16040–16421. The DNA of 19 individuals was extracted and amplified at least twice per individual from different skeletal elements, and the HVR I fragments and other coding region positions were reproduced in several PCR products per extracts. The results of these replicates along with the negative control results suggest the haplotypes are authentic.

The medieval sequences encompass almost the entire range of Western Eurasian macro-haplogroups: H, J, T, R0 and U (S2 Table). However, we observed lower frequencies of haplogroup H and higher frequencies of haplogroups J and T in the medieval population compared with modern-day Europeans, e.g. present-day Slovaks.

The haplogroup frequencies in the medieval population of Slovakia are the following: H 25%, J 20%, T 30% (T1 15% and T2 15%), R0 5% and U 20% (U4 5%, U5a 5% and U8 10%).

Pairwise genetic distances were calculated between 11 medieval populations and between the same medieval populations and a set of modern-day populations from Eurasia. Of the medieval populations, the medieval Poles alone showed non-significant difference from the medieval population of present-day Slovakia (SVK_med) with F_ST_ = 0.02702 and p = 0.08474 ± 0.0025 (S6 Table and Fig 2). Interestingly, pairwise F_ST_ values of modern-day populations indicated a non-significant difference (p > 0.05) only between the medieval Slovakian population and the population from Iraq (F_ST_ = 0.01372, p = 0.08105 ± 0.0081). The present-day Slovaks have a slightly larger genetic distance from medieval Slovaks than other Eastern and Central European modern populations: F_ST_ = 0.05492, p = 0.000 ± 0.000 (S5 Table).

The MDS plot (stress value = 0.1130, S1 Fig) based on linearized Slatkin F_ST_ values contains aggregation of most of the medieval and modern European populations along coordinates 1 and 2; only the Asian populations of Pakistan, Uzbekistan, Kazakhstan and Kyrgyzstan are separated, forming an eastern cluster along coordinate 1. The ancient Xiongnu population from Mongolia is the farthest population along this coordinate. The medieval population from Slovakia is situated along coordinates 1 and 3 among modern European populations, but is differentiated from them along coordinate 2. In contrast, the other medieval European populations are close to modern populations along coordinates 1 and 2, and differentiated along coordinate 3, which implies their stronger affinity to modern European populations compared with the Slovakian dataset.

PCA was calculated based on mtDNA haplogroup frequencies of medieval and modern populations. PCA of medieval populations shows a clustering of populations from Slovakia, Lombards (Hungary) and Poland along the PC1 and PC2 components (39.6% of the variance is displayed) (Fig 3A). The medieval populations and the Vikings of North Europe are clustered together, as well as the medieval populations from South Europe. The farthest populations along the PC1 component are the medieval Mongolians and Cumanians from Hungary, whereas the medieval Hungarians are the utmost population along PC2. Hierarchical clustering shows a very similar diagram to the PCA, except for one phenomenon, namely that the medieval population from Hungary clusters with Lombards from Hungary and with medieval populations from Poland and Slovakia, which we can explain with the interaction of all PCs in this analysis (Fig 3B).

The PCA plots of modern Eurasian populations indicate alignment of the European populations (except Saami) including our investigated medieval population on the PC1 and PC2 components (variance: PC1 = 24.45%, PC2 = 16.60%). However, the different frequencies of haplogroups J, T1, T2, U4, U5a and U8 result in differentiation from all present-day populations along the third component (variance PC3 = 10.71%) (Figs 4 and S2).

The SHA shows that the medieval population from Slovakia shares the majority of haplotypes with medieval populations from Spain and Italy, and that the relatively short genetic distance from the medieval Poles does not mean significant lineage sharing at the HVR I level (S3 Fig).

## Discussion

In this study we investigated a population living in the Hungarian-Slavic contact zone of the Carpathian Basin in the 9^th^–12^th^ centuries AD. Using well established protocols for aDNA analysis, we have obtained the first mtDNA dataset (to the best of our knowledge) concerning the genetic variation of the HVR I region and six nucleotide polymorphisms in the coding region of mtDNA. These markers enabled us to assign the mtDNA haplogroups of the samples.

The representative haplogroups of the investigated medieval population belong to common modern European (West Eurasian) haplogroups. However, their frequencies are quite different from present-day populations. Contrarily, the detected frequency of haplogroup H in the medieval population from Slovakia was lower (25%) than it is in European modern-day populations (including the Slovaks), where this most frequent haplogroup has a frequency of 45.4% [14,15,40]. The most ancient European haplogroup U [41,42] occurs with a similar frequency in the medieval population (20%) and in modern Slovaks (15%) [40]. Haplogroups J and T occurred in the 9–12^th^ centuries two or three times more frequently than in present-day Europe [32,40,43].

The same phenomenon is perceptible in the Lombard population from the western Hungarian site Szólád with regard to the haplogroups H and J, which have either lower (in case of H) or higher (in case of J) frequency compared to other modern European populations [19]. This similarity results in the clustering of the medieval populations of Slovakia and Lombards from Hungary in the PCA (Fig 3). The Lombard people were of diverse origin and their cemetery was in use only for a short period of time in the 6^th^ century. The research team of Alt et al. [19] suggested that people moved as a group whilst also integrating unaffiliated individuals. Based on the observed diversity of the mtDNA haplogroups, they came to the conclusion that this population includes numerous characteristic Central European lineages as well as a few rare haplogroups. Moreover, our PCA, MDS and SHA presented two populations sharing the same cultural label “Lombards” from different locations (Italy and Hungary). Interestingly, they are relatively far from each other in genetic context, in spite of the historical records of Lombard migration across Europe. This probably follows from the characteristic Central European mtDNA lineages of the Lombards from Hungary [19] in contrast to the Lombards from Italy, who had correlation with modern-day populations inhabiting the same Italian geographical area [10].

Furthermore, the PCA showed that the medieval population of Poland clusters with the medieval population of Slovakia and the Lombards from Hungary (Fig 3), which we explain with similar mtDNA haplogroup composition of medieval Poles to the modern Slavic population spread over a major area of Central and Eastern Europe [20]. The medieval Slovakian samples show closer ties with Polish samples of Slavic origin than with 10^th^ century Hungarian samples from Hungary [18]. However, the ongoing analysis of further 10^th^ century samples from the region will probably result in a more accurate estimation of the affinity of the medieval populations.

The medieval population under study shares mtDNA haplogroups with common European populations, which is represented in the PCA of modern Eurasian populations. The connection is especially strong to modern Central and South Europeans (Fig 4).

The MDS plot showed clustering of the medieval population from Slovakia and other European populations especially along the third coordinate, as well as population genetic differentiation from Asia along the first and second coordinates, although some F_ST_ values were not statistically significant (p > 0.05) (S5 Table and S1 Fig).

From the F_ST_ calculation a different result was obtained by the SHA, where a high frequency of shared haplotypes was shown between medieval populations from Slovakia, Spain and Italy. Summarizing, we consider the studied medieval population of Slovakia to be a mixture of groups of different genetic origins. However, more genetic data from Central Europe would be needed to investigate this epoch in detail.

## Conclusion

The comparative analysis of the medieval sample sets suggests that the present-day European gene pool could have been formed in the medieval era. This is supported by the presence of a majority of haplogroups and haplotypes in the medieval period which still exist in modern-day Europe. Genetic maternal lineages of the medieval population of present-day Slovakia were diverse and rather similar to medieval Lombards from Hungary and to medieval Slavs from Poland, as well as to modern Europeans, which supports the historical and archaeological standpoint of mixed populations of medieval Slavs and Magyars in the cemeteries investigated. However, the results could have been biased due to the small sample size, and genetic drift could also unnoticeably influence our results. Furthermore, we assessed population interactions and migrations only along the maternal lineages; the paternal side of the medieval population history remained unexamined in this study.

The collection of archaeological relics from genetically examined Slovakian cemeteries overlaps the image drawn by population genetics analysis. The finds of the 10^th^ century cemeteries originated in the material culture of the preceding era, implying that Hungarian groups arriving in the territory blended in with the local population, genetically as well as culturally; this interaction produced the medieval population of the area.

Undoubtedly, further research, including the analysis of the whole mitochondrial genome and nuclear DNA markers, is necessary in the investigation of the genetic connections between medieval and modern populations and in the clarification of the genetic origin of Slavs and Hungarians. Nevertheless, the study presented here of the first successful description of medieval mtDNA variability from Slovakia contributes an important dataset to help reveal the genetic diversity of medieval Europe.

## Supporting Information

S1 Fig MDS plot of medieval and modern European populations. The MDS plot was performed with linearized Slatkin F_ST_ values of 12 medieval and 32 modern Eurasian populations. Its stress value is 0.1130.
(TIF)

S2 Fig PCA of the investigated medieval population and modern-day populations. The PCA is based on mtDNA haplogroup frequencies of the medieval population from Slovakia and 33 modern-day populations from Eurasia, and shows PC1 and PC3. The haplogroup frequencies and the population information are shown in S8 Table.
(TIF)

S3 Fig Levelplot of shared haplotype analysis (SHA). The levelplot is based on the percentage values of the relative shared haplotypes, which are also shown on the figure. The absolute values and the population information are given in S9 Table.
(TIF)

S1 Table Information about the Samples. (XLSX)

S2 Table Haplotypes and haplogroups of samples. Haplotypes based on rCRS and RSRS, range of HVR I, haplogroup-diagnostic positions of mtDNA coding region and haplogroup definitions of the investigated samples used in statistical analysis.
(XLSX)

S3 Table PCR primer pairs. PCR primer pairs used for amplification of HVR I sequences and some SNPs in the coding region of mtDNA.
(XLSX)

S4 Table References of published mtDNA datasets used for statistical analysis. (XLSX)

S5 Table F_ST_ values, p values and Slatkin matrix of medieval and modern populations. F_ST_ values, p values and Slatkin matrix of 12 medieval populations and 32 modern populations from Eurasia.
(XLSX)

S6 Table F_ST_ values, p values and Slatkin matrix of medieval populations. F_ST_ values, p values and Slatkin matrix of 11 medieval populations from Europe and one population from Asia.
(XLSX)

S7 Table MtDNA haplogroup frequencies of medieval populations. Population information and mtDNA haplogroup frequencies used for PCA with 12 medieval populations (11 from Europe and one from Asia).
(XLSX)

S8 Table MtDNA haplogroup frequencies of modern-day populations. Population information and mtDNA haplogroup frequencies used for PCA with the medieval population from Slovakia and 33 modern-day Eurasian populations.
(XLSX)

S9 Table Shared haplotype analysis (SHA) of medieval populations. The frequencies of shared haplotypes of 12 medieval populations.
(XLSX)

S10 Table HVR I motifs of the researchers. HVR I haplotypes of the researchers who had been in contact with the ancient samples during the laboratory work.
(XLSX)
