# Supplementary figures and images for: Maternal Genetic Composition of a Medieval Population from a Hungarian-Slavic Contact Zone in Central Europe
Source: PLoS One. 2016 Mar 10;11(3):e0151206. doi: 10.1371/journal.pone.0151206 (PMC4786151; doi:10.1371/journal.pone.0151206)

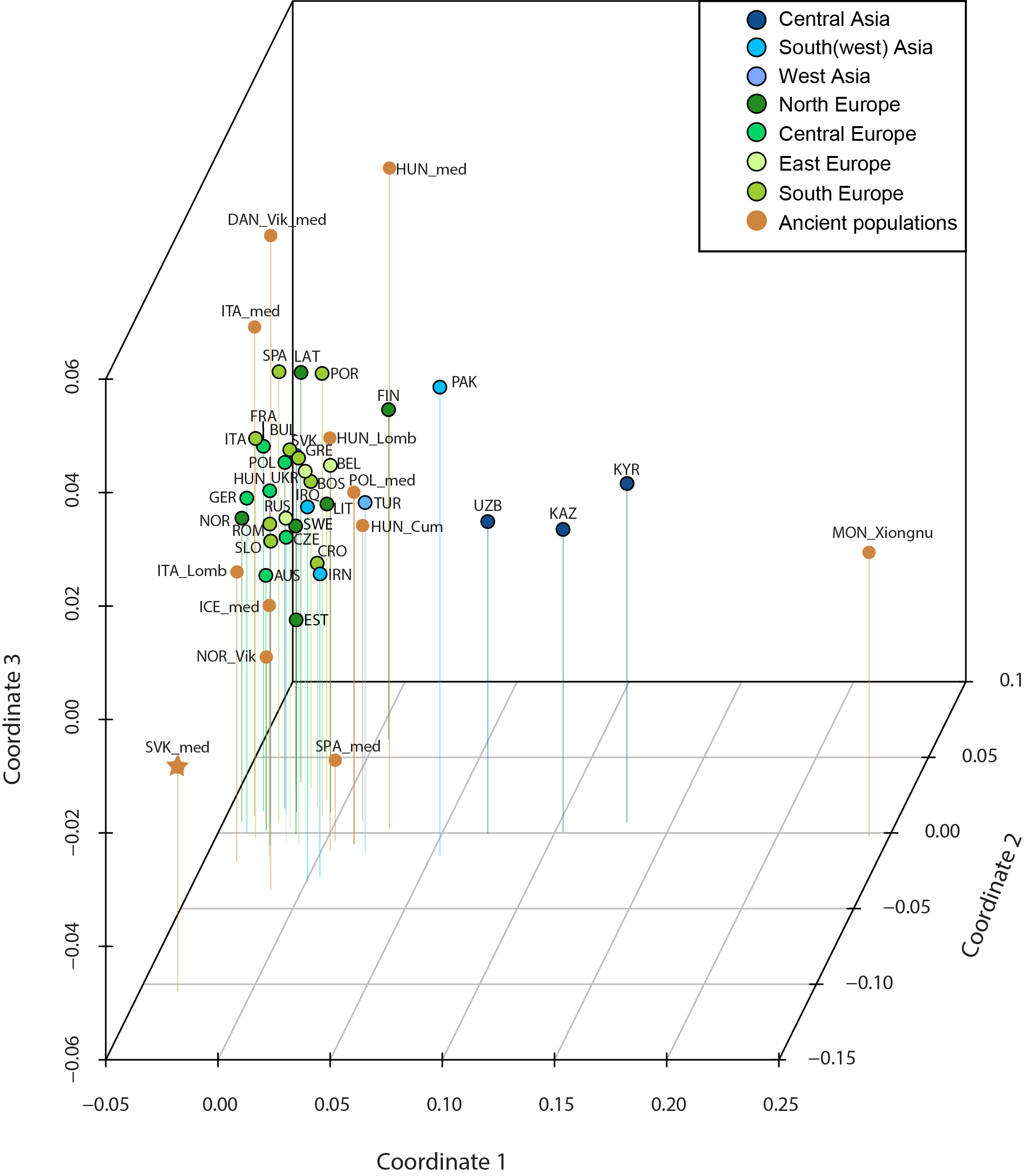

Supplement: S1 Fig — The MDS plot was performed with linearized Slatkin FST values of 12 medieval and 32 modern Eurasian populations. Its stress value is 0.1130. (TIF) [file pone.0151206.s001.tif]

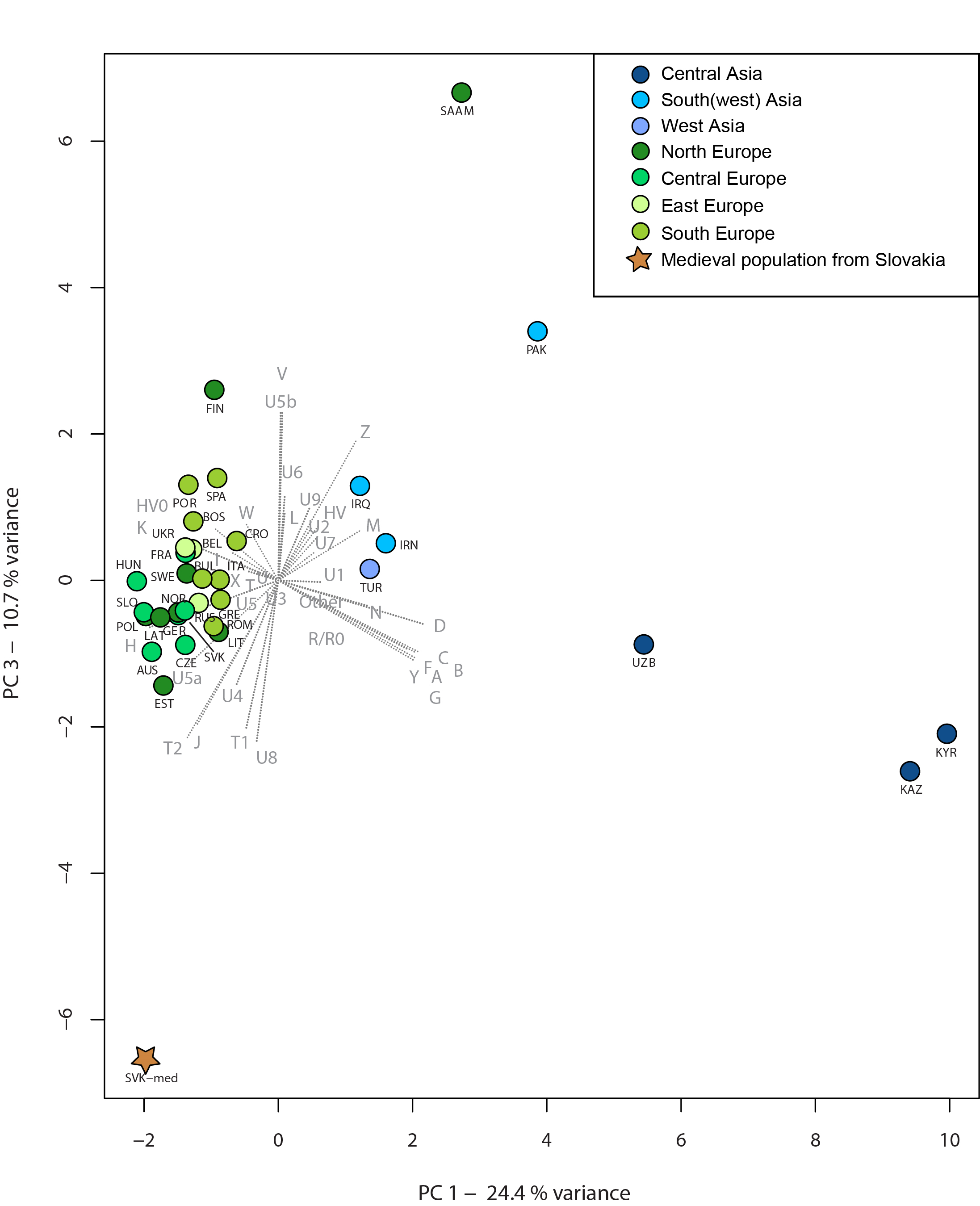

Supplement: S2 Fig — The PCA is based on mtDNA haplogroup frequencies of the medieval population from Slovakia and 33 modern-day populations from Eurasia, and shows PC1 and PC3. The haplogroup frequencies and the population information are shown in S8 Table. (TIF) [file pone.0151206.s002.tif]

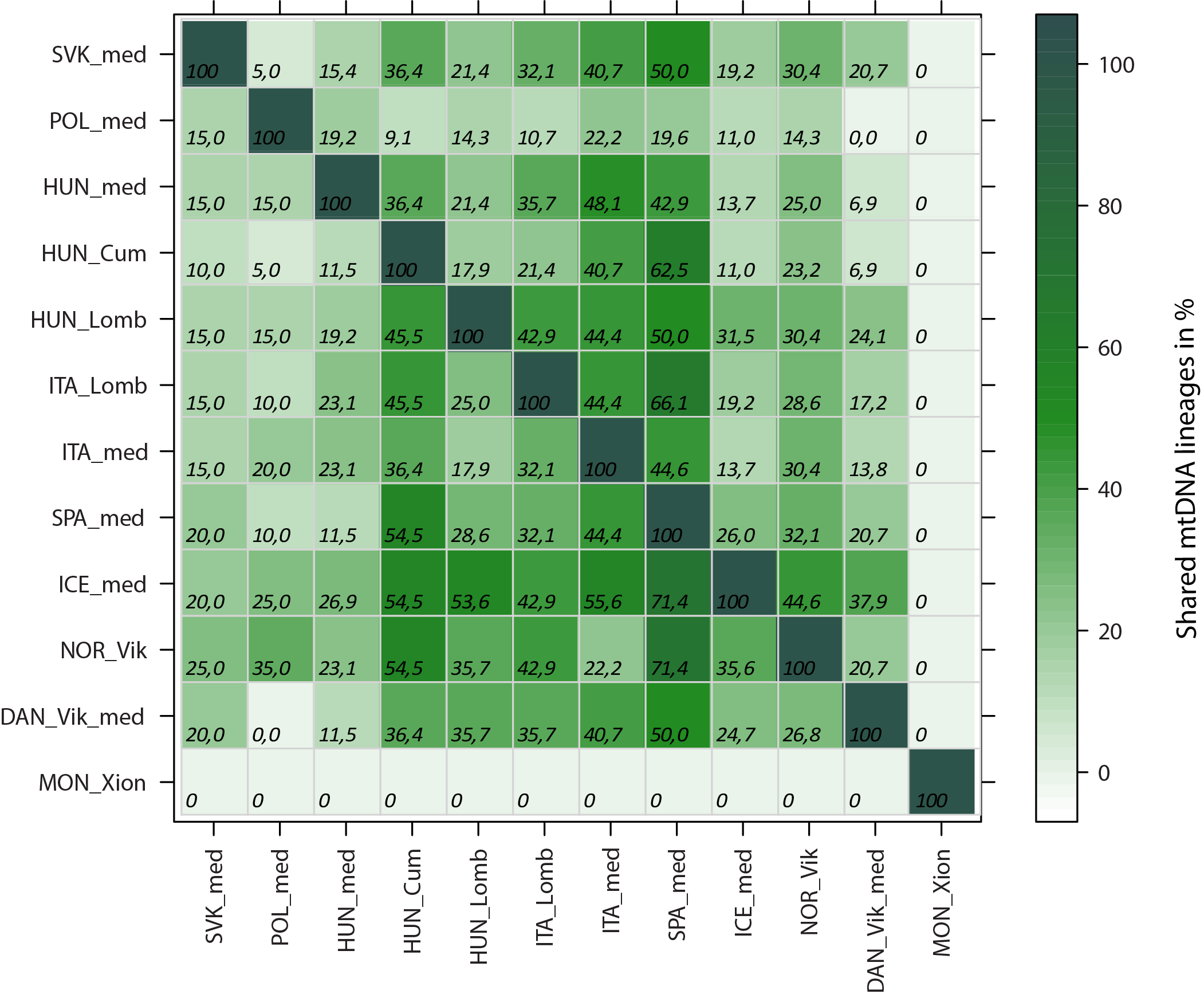

Supplement: S3 Fig — The levelplot is based on the percentage values of the relative shared haplotypes, which are also shown on the figure. The absolute values and the population information are given in S9 Table. (TIF) [file pone.0151206.s003.tif]
